# Enzyme engineering for functional lipids synthesis: recent advance and perspective

**DOI:** 10.1186/s40643-023-00723-7

**Published:** 2024-01-02

**Authors:** Ailin Guan, Yue Hou, Run Yang, Jiufu Qin

**Affiliations:** College of Biomass Science and Engineering, Sichuan University, Chengdu, 610065 China

**Keywords:** Functional lipids, Biocatalysis, Protein engineering, Activity, Selectivity, Stability

## Abstract

Functional lipids, primarily derived through the modification of natural lipids by various processes, are widely acknowledged for their potential to impart health benefits. In contrast to chemical methods for lipid modification, enzymatic catalysis offers distinct advantages, including high selectivity, mild operating conditions, and reduced byproduct formation. Nevertheless, enzymes face challenges in industrial applications, such as low activity, stability, and undesired selectivity. To address these challenges, protein engineering techniques have been implemented to enhance enzyme performance in functional lipid synthesis. This article aims to review recent advances in protein engineering, encompassing approaches from directed evolution to rational design, with the goal of improving the properties of lipid-modifying enzymes. Furthermore, the article explores the future prospects and challenges associated with enzyme-catalyzed functional lipid synthesis.

## Introduction

Functional lipids are increasingly recognized for their potential to impart health benefits, ranging from cardiovascular health to mental well-being and metabolic regulation, including diabetes management (Wu et al. 2022a). The synthesis of these bioactive lipids involves the modification of natural lipid structures through methods such as chemical reactions and enzymatic catalysis (Biermann et al. 2021; Bornscheuer 2018). This process yields functional lipid derivatives, including diacylglycerols (DAGs), structured triglycerides (TAGs), and structural phospholipids (PLs), precisely tailored to meet specific nutritional requirements (McDaniel et al. 2003; Xu et al. 2023). However, conventional chemical methods often require rigorous reaction conditions and yield significant byproducts, posing challenges for the efficient synthesis of desired functional lipids.

In contrast to chemical methods, enzymes stand out as biodegradable biocatalysts, offering significant advantages in the industry, such as high selectivity, mild operating conditions, and reduced byproduct formation (Madhavan et al. 2021). Structured TAGs undergo hydrolysis, esterification, or interesterification catalyzed by enzymes such as lipases and phospholipases, with a primary focus on modifying the composition and/or position of fatty acids in lipids. This intricate process leads to the synthesis of a diverse array of functional lipids to meet specific nutritional demands, including medium-long-medium structured lipids (MLM-SLs), human milk fat substitutes (OPO, OPL), DHA-enriched TAGs, and various other structural lipids (Zhu et al. 2023; Zorn et al. 2016; Zou et al. 2020). Additionally, structured PLs, subject to modification by enzymes like phospholipase A2 and phospholipase D, encompass DHA/EPA-enriched phospholipids and derivatives with distinct head groups, such as saccharides, phenylalkanols, terpenes, and ethanolamine derivatives (Hayashi et al. 2021; Zhang et al. 2019b; Zhang et al. 2020b).

**Fig. 1:**
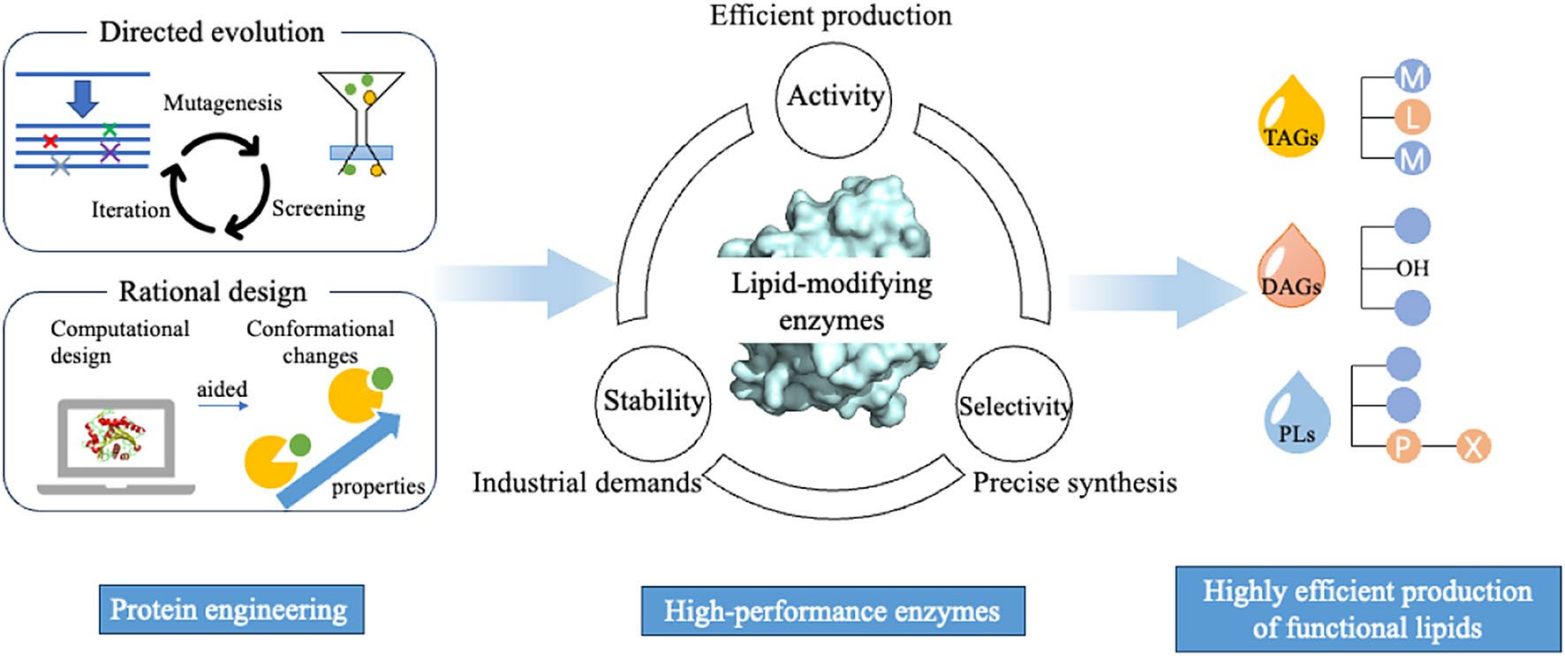
Protein engineering, encompassing directed evolution and rational design, has been utilized to enhance the performance of lipid-modifying enzymes in the synthesis of functional lipids. The products include, but are not limited to: (1) Triglycerides (TAGs), such as medium-long-medium structured lipids (MLM-SLs); (2) Diacylglycerides (DAGs); and (3) Phospholipids (PLs) with various acceptor alcohols X (e.g., saccharides, phenylalkanols, terpenes, and ethanolamine derivatives)

The suboptimal performance of enzymes and high costs remain challenges in the enzymatic modification of functional lipids. Recently, significant progress in protein engineering has evolved, shifting from random mutagenesis techniques (Elizabeth 2022; Yang and Arnold 2021) to more targeted rational design approaches (Madhavan et al. 2021; Reetz et al. 2005; Reetz and Carballeira, 2007). Protein engineering involves the modification of sequences in natural proteins with the goal of enhancing their activity(Lovelock et al. 2022; Wu et al. 2022c) and stability (Adi Goldenzweig 2018; Li et al. 2022c), and potentially optimizing or altering their selectivity (Wu et al. 2022b; Zheng et al. 2023), thereby creating tailored enzymes. Recent advancements in machine learning (Lu et al. 2022; Mazurenko et al. 2019) and artificial intelligence, such as AlphaFold (Varadi et al. 2022), Rosetta-Fold (Baek et al. 2021; Watson et al. 2023) have emerged as promising approaches directing protein engineering. This manuscript serves as a review of advanced technologies in protein engineering, encompassing directed evolution and rational design for modifying enzyme activity, selectivity, and stability, within the context of developing functional lipids (Fig. 1).

## Enhancing functional lipid synthesis via protein engineering

### Improving enzyme activity for the high-yield synthesis of functional lipids

In the realm of industrial biocatalysis, enhancing enzyme activity for specific substrates is a critical factor for improving production efficiencies. This is especially pertinent in the synthesis of specific lipids, where improved enzymatic activity leads to more effective and precise lipid processing.

Mimicking natural evolution, directed evolution systematically enhances enzyme properties by improving specific protein traits through multiple rounds of mutation and screening (Arnold 2018; Kuchner 1997). The advantage of directed evolution lies in its ability to obtain desired enzyme variants with limited knowledge of protein structure information and catalytic mechanisms. This approach has found wide application in lipid-modifying enzymes, including lipase (Zhang et al. 2020a), phospholipase D (PLD) (Zhang et al. 2019a), and oxidative fatty acid decarboxylases (OleT) (Markel et al. 2021). For instance, to enhance the transphosphatidylation activity of PLD, a directed evolution approach was employed, utilizing DNA shuffling and an autodisplay system for efficient mutant screening. This strategy identified three beneficial mutations in PLD, with the top-performing mutation demonstrating an 80.3% phosphatidylserine content and a 3.24-fold increase in transphosphatidylation conversion compared to the wild type (WT). The study also emphasized the influence of C-terminal amino acids on PLD folding and underscored the significance of N-terminal amino acids in catalytic reactions (Zhang et al. 2019a). Although directed evolution is a powerful method for enzyme modification, the challenge lies in the vastness of the mutation library, resulting in significant screening pressure (Bornscheuer et al. 2019; Qu et al. 2020). The development of efficient screening methods is crucial to enhance its effectiveness (Zeng et al. 2020).

On the basis of protein sequence information, structural details, and catalytic mechanisms, the catalytic activity or selectivity modification of enzymes primarily targets residues within the substrate-binding pocket or channel (Yu et al. 2019; Zheng et al. 2023). This involves reshaping the volume of the substrate-binding pocket to accommodate substrates appropriately, eliminating spatial conflicts in the molecular channel to facilitate substrate transfer, or directly optimizing the enzyme–substrate interaction. These rational strategies contribute to a certain extent in reducing screening costs, but their success is significantly influenced by the careful selection of mutation targets. This necessitates a thorough understanding and awareness of protein information by researchers (Table 1).

**Table 1:** Advancements in enzyme engineering for functional lipids synthesis

Properties	Enzymes	Applications	Engineering strategies	Performance	Reference
Activity	Phospholipase D (PLD)	For the enzymatic production of phosphatidylserine	Directed evolution	The mutation demonstrating a 3.24-fold increase in transphosphatidylation conversion compared to the WT	(Zhang et al. 2019a)
	PLD	For the enzymatic production of phosphatidylserine	Substrate pocket reconstruction strategy	The mutant displayed 2.04-fold increase in the transphosphatidylation/hydrolysis ratio compared to the WT	(Qi et al. 2022)
	*Candida antarctica* lipase A (CALA)	For the enrichment of long chain mono-unsaturated fatty acids	Reshaping of binding tunnels	The variant V290W doubled C20:1 in the esterified fraction from 15 to 34%	(Zorn et al. 2019, 2018)
Selectivity	Lipase MAS1	For the enzymatic production of DAGs	Substrate binding pocket engineering	The mutation showed an increased synthesis ratio of partial glycerides/triglycerides to 6.32, compared to 1.21 in the WT	(Yang et al. 2022)
	*Candida antarctica* lipase B (CALB)	For the enzymatic production of 1-monoacyl-sn-glycerol	Substrate binding pocket engineering	The mutation showed twofold increase in selectivity for synthesizing 1-monoacyl-sn-glycerol	(Woo et al. 2022)
	PLD	For PLD selectivity, the positional specificity toward the 1-OH of myo-inositol	Substrate binding pocket engineering	The mutation showed remarkable 98% positional specificity	(Samantha et al. 2021)
	Fatty acid hydratases	For the enzymatic production of high-value HFAs	Sequence alignment and structure analysis	The mutation shifted the ratio of the HFA regioisomers (10-OH/13-OH) from 99:1 to 12:88	(Eser et al. 2020)
	Lipoxygenases (LOX)	For the enzymatic production of 13R-hydroxy-docosahexaenoic acid and 13R,20-dihydroxy-docosahexaenoic acid from DHA	Catalytic mechanism-based site-directed mutagenesis	The catalytic properties of the mutant have shifted from 13S-LOX to 9R-LOX	(Yi et al. 2020)
Stability	*Rhizopus oryzae* lipase (ROL)	For the enzymatic production of TAGs	Sequence alignment	The mutant retains most of its activity at 70 °C, whereas the WT is incapable of functioning at temperatures above 60 °C	(Chow and Nguyen 2022)
	*Yarrowia lipolytica* lipase Lip2	For the enzymatic production of MLM-SLs	Molecular dynamic (MD) simulation and the introduction of disulfide bonds	The mutant 4sN exhibited an increase in stability, with a rise in melting temperature (*T*_m_) of 19.22 °C	(Li et al. 2022b)
	Phospholipase C (PLC)	For enzymatic degumming of vegetable oils	B-factor analysis and MD simulation	The mutation F96R/Q153P showed the highest optimal reaction temperature (90 °C)	(Zhang et al. 2022b)
	PLD	For the enzymatic modification of phospholipids	Disulfide bond engineering	The mutation showed a 3.1-fold increase in half-life (*t*_1/2_) at 35 °C and a 5.7 °C rise in *T*_m_	(Li et al. 2022a)

**Fig. 2:**
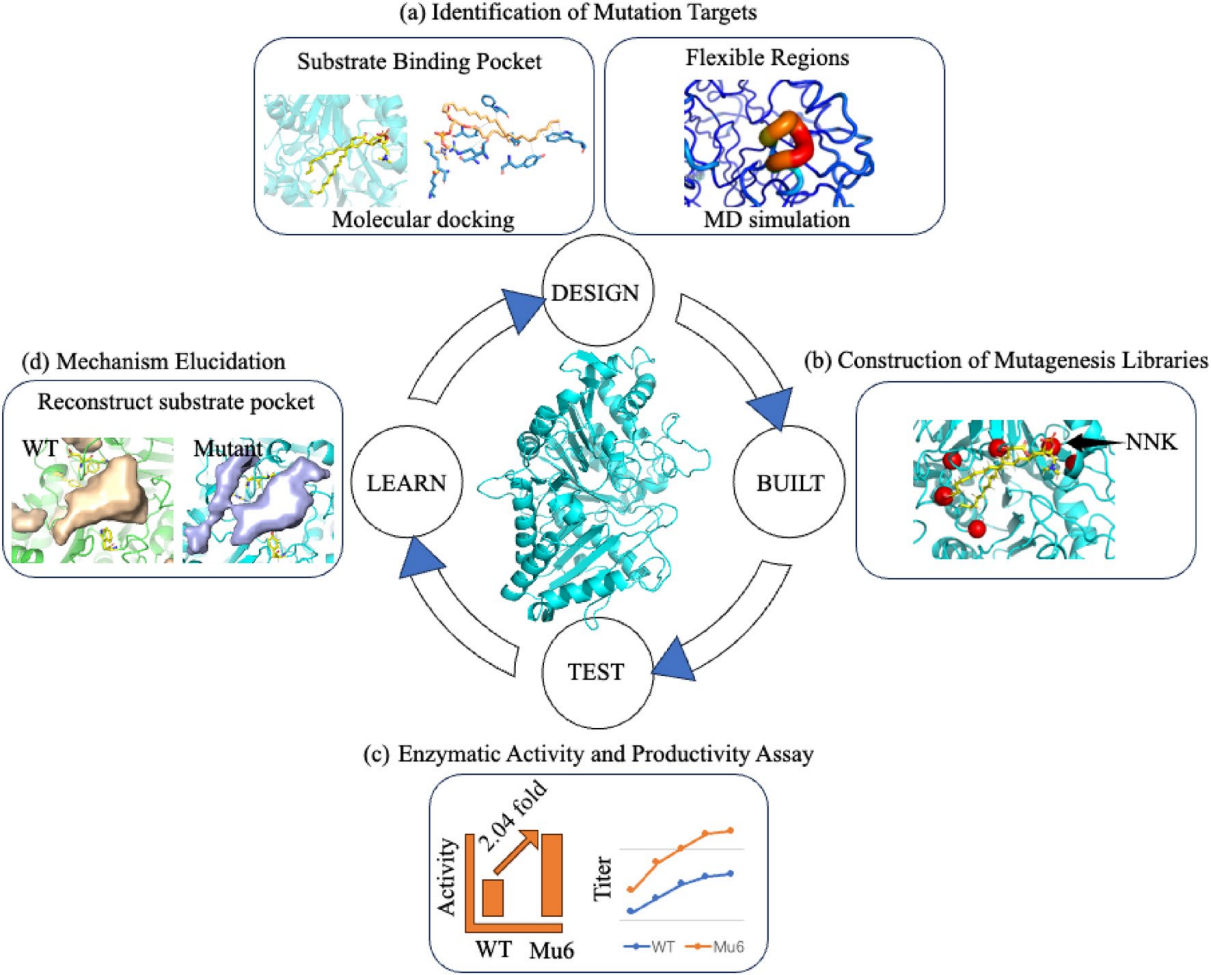
Schematic representation of rational design to reconstruct the substrate pocket for improved PLD activity in phosphatidylserine production. The PLD structure depicted in **a**, **b**, **c** was modeled utilizing SWISS MODEL, utilizing the template protein from Protein Data Bank (PDB) ID: 1f0i and visualized using PyMOL; AutoDock Vina was employed to perform docking of PLD in complex with the substrate as shown in (**a**), with subsequent analysis conducted using the PLIP server; **c** Experimental assessment of enzyme activity and phosphatidylserine synthesis on a 3-liter scale; **d** Hydrophobic cavity computed with the POCASA server, indicating changes before and after mutations, implying an expanded substrate pocket for better substrate accommodation as one of the reasons for enhanced activity. Detailed experimental results are provided in the reference (Qi et al. 2022)

One such strategy, termed "substrate pocket reconstruction" guided by insights from the catalytic mechanism, involved expanding the substrate-binding pocket and making precise adjustments in the coordination of the substrate within the active site (Fig. 2). Molecular docking provided information on the force network between the enzyme and substrate, combined with molecular dynamics (MD) to calculate critical distances between catalytic residues and the substrate, such as nucleophilic attack distance. Simultaneously, MD simulations revealed that flexible regions in the "top" loop tended to approach the active site. These analyses indicated the direction for target mutation. About 28 residues near the active site and flexible regions were identified as potential mutation sites for NNK site-saturation mutagenesis (SSM). The resulting optimal mutant displayed a notable 2.04-fold increase in the transphosphatidylation/hydrolysis ratio compared to the WT. Under optimal conditions, the mutant Mu6 achieved a production of 58.6 g/L of phosphatidylserine with a 77.2% conversion within 12 h on a 3 L scale, showcasing its potential for industrial application (Qi et al. 2022). In another case, a conserved flexible loop (residues 376–382) in the active site of *Streptomyces klenkii* PLD (SkPLD) was identified based on sequence conservation and amino acid analysis. Mutating the only hydrophilic residue Ser380 to Val in this loop resulted in a 4.8-fold increase in catalytic efficiency and nearly seven times higher adsorption equilibrium coefficient compared to the wild-type SkPLD. The findings indicate that the loop containing residue S380 in SkPLD plays a crucial role in interfacial binding and substrate recognition (Hu et al. 2021).

**Fig. 3:**
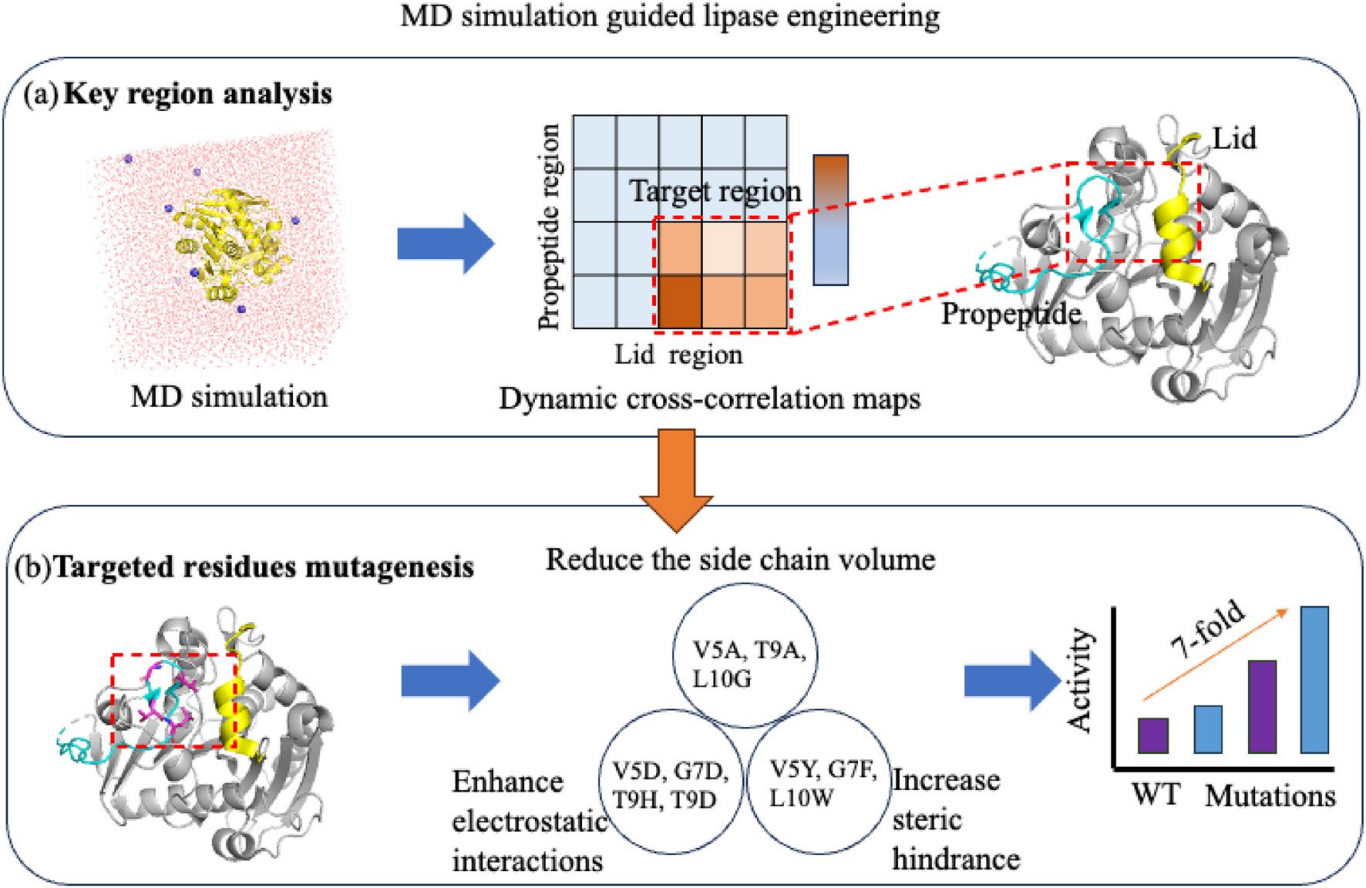
Schematic representation of MD simulation guided rational design for improving lipase activity. **a** Visually represents the application of Dynamic Cross-Correlation (DCC) analysis, based on MD simulations, to identify the critical interaction area between the propeptide and the lid. (**b**), the impact on hydrolysis activity is demonstrated through the introduction of mutations via site-directed mutagenesis, organized into three distinct groups. Additional methodologies, including Principal Component Analysis (PCA) and Interaction Graph Modeling (IGM), were also utilized to discern the movement pattern of the propeptide and pinpoint the critical interaction area with the lid. Further detailed information is available in the referenced literature (Wang et al. 2021)

Based on a comprehensive understanding of lipase structural characteristics, lid dynamics, and the roles played by lids in lipase catalysis, lipases have been the subject of extensive protein engineering efforts (Chen et al. 2022; Ge et al. 2023; Maldonado et al. 2021; Soni 2022). The phenomenon of lipase interfacial activation, characterized by a significant increase in activity at the interface between oil and water, is intricately linked to a distinct domain in lipases known as the "lid" (Verger 1997). Site-directed mutagenesis was utilized to target hydrophobic residues in the lid region of T1 lipase, replacing them with hydrophilic counterparts. Notably, mutants A186S and A190S displayed a 35–50% increase in catalytic efficiencies compared to the WT, while retaining their functionality at elevated temperatures (Tang et al. 2017). In recent times, a distinct mono- and diacylglycerol lipase (MDGL) derived from the fungus *Aspergillus oryzae* has become a focal point in academic discussions. The crystal structure of Aspergillus oryzae lipase (AOL) has been successfully resolved at a resolution of 1.7 Å. Analysis of the structure and alignment of AOL with other MDGLs unveiled the critical role of residue V269 in catalysis. Following this discovery, the engineered variant V269D demonstrated a hydrolysis activity approximately 6 times higher than that of the WT (Lan et al. 2021). Besides, recent studies have identified the propeptide region of lipase as a potential target for engineering modifications. Taking *Rhizopus chinensis* lipase (RCL) as an example, analysis through MD simulations of the enzyme–substrate complex revealed that the propeptide uncovered a crucial region (Val5-Leu10), inhibiting the movement of the lid (Fig. 3). Mutations in this region significantly increased catalytic efficiency by 700% (Wang et al. 2021).

### Tailoring enzyme selectivity for the precise synthesis of functional lipids

A diverse range of enzymes is applicable for the modification of fats, oils, and other lipids due to their inherent excellent chemo-, regio-, and stereoselectivity (Bornscheuer 2014). Although exploring lipid-modifying enzymes like lipases in nature for specific selectivities can be challenging and not always fruitful (Maldonado et al. 2021), a viable alternative is the modification of existing lipases through protein engineering techniques.

Similar to the modification of enzyme activity, the targeted mutations for selective modification are primarily focused on the substrate binding pocket. Some intriguing examples suggest that even a single or double residues mutations in this area has the potential to alter the selectivity of lipase. For instance, the substitution of TAGs with diacylglycerides (DAGs) has been demonstrated to effectively reduce body fat accumulation and aid in weight loss (Prabhavathi Devi et al. 2018). However, despite purification efforts, the obtained DAG level remains less than 60%, and high levels of by-products, such as monoacylglycerides (MAGs) and free fatty acids (FFAs), are observed (Lee et al. 2020; Xu et al. 2023). Consequently, structural analysis of lipase MAS1 suggests that the charge and steric hindrance associated with the T237 residue at the entrance of the substrate-binding pocket may influence substrate binding or product release. The T237R mutation resulted in an increased synthesis ratio of partial glycerides/triglycerides to 6.32, compared to 1.21 in the WT. Changes in hydrophobicity and steric hindrance contributed to a higher production of DAG over TAG. This highlights the significant potential of the T237R mutation as a mono- and diacylglycerol lipase (Yang et al. 2022). Likewise, directing attention to the substrate-binding region of *Candida antarctica* lipase B (CALB), the engineered variants CALB_A282E/I285F_, designed with a constrained substrate binding region, exhibited an approximate twofold improvement in selectivity for the synthesis of 1-monoacyl-sn-glycerol with n-nonanoic acid. The resultant double mutant facilitated the generation of 1-nonanoyl-glycerol, achieving a concentration of 2.27 M in glycerol, with a reaction rate of 1.0 M/h (Woo et al. 2022). Furthermore, molecular docking and computational simulations, calculating substrate-enzyme binding energy, assess the absolute affinity between lipases and TAGs, offering theoretical guidance for rational lipase selectivity design (de Rodrigues et al. 2021).

Furthermore, a significant proportion of natural PLs can undergo synthesis via PLD-mediated transphosphatidylation involving phosphatidylcholine (PC) and corresponding alcohols. Nevertheless, the enzyme exhibits selectivity for alcohols, thereby constraining the molecular size of acceptor compounds and limiting the range of synthesizable phospholipid species. To enhance the positional specificity of engineered PLD toward the 1-OH of myo-inositol, a comprehensive three-round mutagenesis approach was systematically implemented, targeting residues within the substrate-binding site of PLD. The most successful variant demonstrated exceptional positional specificity, reaching up to 98%. This investigation shows promise for expanding the substrate spectrum of PLD and facilitating the synthesis of diverse phospholipid species (Samantha et al. 2021). Additionally, engineering of a PLD for the purpose of enzymatically producing "difficult-to-synthesize" PLs, such as phosphatidylthreonine (Damnjanović et al. 2018) and 1-phosphatidyl-β-D-glucose (Inoue et al. 2016), has proven to be effective.

The trans fatty acids, considered undesirable constituents of unsaturated fatty acids, can have notable adverse effects on human health. These effects include the potential to induce heart disease or metabolic dysfunction (Aldai et al. 2013; Micha and Mozaffarian 2009). The fatty acid photo-decarboxylase (FAP) is acknowledged for its effectiveness in catalyzing the decarboxylation of trans fatty acids, producing readily-removed hydrocarbons and carbon dioxide. However, there is a need to enhance its selectivity for trans fatty acids while leaving cis fatty acids unchanged. To address this, a highly effective protein engineering strategy, known as "focused rational iterative site-specific mutagenesis," was employed to improve the selectivity of the photo-decarboxylase. The optimal mutant V453E exhibited a remarkable one-thousand-fold improvement in trans-over-cis selectivity compared to the WT. This improvement was attributed to the reinforced electronic interaction between the enzyme's residues and the double bond of the substrate, thereby stabilizing the binding of elaidic acid in the channel (Li et al. 2021).

Hydroxy fatty acids (HFAs) represent distinctive fatty acid (FA) derivatives known for their beneficial medical properties (Bergamo et al. 2014; Ogawa 2015). This category includes branched FA esters of HFAs (FAHFAs) and specialized pro-resolving mediators (SPM) which exhibit effects such as antidiabetic, inflammation resolution, and tumor growth suppression (Sulciner et al. 2018; Yore et al. 2014). The enzymatic conversion of FAs using fatty acid hydratases (FAHYs) provides an environmentally friendly pathway for HFA production. However, the diversity of HFAs generated has been historically limited in terms of chain length and hydroxy position. A rational design approach, guided by a comparative analysis of enzyme active sites, led to the development of a three-residue mutant of FAHY that exhibited a notable reversal of regioselectivity towards linoleic acid. This mutation shifted the ratio of HFA regioisomers (10-OH/13-OH) from the original 99:1 to a new distribution of 12:88 (Eser et al. 2020). In addition to the utilization of FAHYs, cytochrome P450 monooxygenases (CYPs) are employed for the enzymatic hydroxylation of fatty acids, resulting in valuable HFAs. However, CYPs typically yield complex mixtures of HFA regioisomers. To overcome this limitation, extensive site-directed and site-saturation mutagenesis techniques were applied to isolate variants with high regioselectivity. These mutants demonstrated the capability to selectively produce a single HFA regioisomer (either ω-1 or ω-2) with selectivity ranging from 75 to 91% when using fatty acids ranging from C12 to C18. This makes them promising candidates for the production of pure HFA isomers (Zong et al. 2023).

### Improving enzyme stability to adapt to the processes in the lipid modification industry

The application of enzymes in the industrial synthesis of functional lipids may face challenges associated with high temperatures. For instance, elevated processing temperatures can simplify industrial degumming processes by reducing oil viscosity and mitigating microbial contamination, thereby facilitating enzymatic hydrolysis (Zhang et al. 2022b). The utilization of more thermostable enzymes in the industry has shown several benefits, such as improved biocatalytic efficiency, shortened processing times, and ultimately reduced energy consumption (Rathi et al. 2016). Protein engineering has made significant contributions to the thermal stability modification of enzymes. Currently employed methods for enhancing enzyme thermal stability include disulfide bond “stapling”, B-factor engineering, conformational free energy calculations, and N-terminal domain substitution.

Firstly, the introduction of covalent bonds, such as disulfide and thioether bonds, has been shown to enhance enzyme thermal stability. Identification of flexible regions in lipase Lip2 through MD simulation led to the subsequent engineering of disulfide bonds into these regions, resulting in the creation of the mutant 4sN. This mutant exhibited significant improvements in both melting temperature (*T*_m_) and the half-loss temperature at 15 min (*T*^15^_50_), with enhancements of 19.22 °C and 27.75 °C, respectively. To assess the practical utility of mutant, the performance in synthesizing MLM-SLs using immobilized mutant 4sN has been further evaluated. At 12 h, mutant 4sN achieved incorporations of 18.24% and 20.43% at 40 °C and 45 °C, respectively, surpassing the wild type, which remained below 15% (Li et al. 2022b). Furthermore, by employing the Rosetta Cartesian_ddg protocol to calculate changes in conformational free energy, potential mutations were predicted to enhance the stability of *Rhizopus oryzae* lipase (ROL). Through site-directed mutagenesis and the introduction of disulfide bonds, a variant exhibited improved stability, with an 8.5 °C increase in *T*_m_ and a half-life of 31.7 min at 60 °C, representing a 4.2-fold increase compared to the WT. Subsequently, the investigation evaluated the capacity of mutants to generate FAs from tricaprin and soybean oil under equivalent enzyme protein concentrations. After 12 h, the mutant demonstrated a notable hydrolysis rate of 97.2%, outperforming the WT, which achieved only 78.7%. This disparity underscores the positive impact of increased thermostability on catalytic efficiency (Huang et al. 2023). Additionally, techniques such as B-factor analysis based on structural biology, have been widely employed to enhance the thermal stability of enzymes (Sun et al. 2019). The amino acid residues crucial for thermal stability, often located in flexible protein regions, is achieved through B-factor analysis. For Phospholipase C (PLC), a rational design strategy incorporating B-factor analysis and MD simulation was employed. The resulting variant, F96R/Q153P, exhibited a notable increase in its optimal reaction temperature (90 °C) and 2.37-fold enhancement in *k*_cat_/*K*_m_. The mutant shows great potential application in food processing industries such as enzymatic degumming under extreme high temperature(Zhang et al. 2022b). Substituting the N-terminal structural domain is another method for enhancing enzyme thermal stability, and this modification often brings surprising changes to the enzyme's functionality. To illustrate, a monoglyceride lipase (TON-LPL) from the hyperthermophilic archaeon *Thermococcus onnurineus* was selected and successfully transformed it into a triglyceride lipase using a N-terminal domain substitution approach. As anticipated, the mutant exhibited thermal stability, displaying optimal temperature at 60 °C, along with the desired enzymatic activity (Soni et al. 2019).

## Conclusion and outlook

Despite substantial efforts in protein engineering aimed at modifying industrial enzyme catalysts, a gap persists between advancements at the laboratory level and large-scale production. In recent years, significant contributions to the field have been made by immobilization techniques for lipid-modifying enzymes (Ahrari et al. 2022; Akil et al. 2020; Enespa et al. 2022; Martins et al. 2022; Pacheco et al. 2022; Verdasco-Martín et al. 2018; Zhang et al. 2022a). Free enzymes show low operational stability, have high costs, and cannot be easily recovered or reused at the end of the reaction, hindering product separation (Almeida et al. 2021). Immobilization techniques aim to anchor enzymes on solid supports, employing various methods such as adsorption, entrapment, covalent bonding, and cross-linking, to enhance biocatalyst stability and facilitate recovery/reuse steps. However, the work of protein engineering contributes to creating enzymes more suitable for immobilization. For example, enzyme immobilization often achieves reusable biocatalysts with improved operational stability and solvent resistance, but this is often accompanied by some loss of enzyme activity (Bernal et al. 2018). Protein engineering is used to provide enzymes with higher performance to compensate for these losses. Changes induced in enzymes through protein engineering may enhance their affinity, allowing them to adapt to specific immobilization carriers. Additionally, altering the enzyme's surface properties through protein engineering may improve its stability, activity, or selectivity in the immobilized state. Besides, given the intrinsic complexity of lipase/phospholipase catalytic systems, which differ from homogeneous enzyme catalytic systems, there is a need for engineering the reaction medium (Cao et al. 2022; Wang et al. 2023). The integration of multiple technologies, including protein engineering, immobilization techniques, and reaction medium engineering, represents a method to extend the industrial application of enzyme-mediated lipid modification.

Moreover, a notable transformation is occurring in the realm of biocatalysis for lipid modification, particularly in the context of whole-cell biocatalysts. Diverse microorganisms, including bacteria, yeast, fungi, and microalgae, exhibit the capability to biosynthesize fatty acids utilizing a range of raw materials such as glucose, cellulose, starch, glycerol, and even one-carbon compounds. The evolution of synthetic biology has provided the means to construct microbial cell factories. These cell factories, rooted in the principles of metabolic engineering, serve as platforms for microbial synthesis, enabling the production of targeted compounds (Nielsen and Keasling 2016). For instance, a *Saccharomyces cerevisiae* platform was engineered for the de novo synthesis of oleoylethanolamide, a phospholipid derivative with significant potential in pharmacological applications for mitigating lipid dysfunction and neurobehavioral symptoms (Liu et al. 2020).

## Data Availability

All relevant data supporting the findings of this study are available within the article. Additional data are available from the corresponding author upon reasonable request.

## References

[CR1] Adi Goldenzweig SJF (2018). Principles of protein stability and their application in computational design. Annu Rev Biochem.

[CR2] Ahrari F, Yousefi M, Habibi Z, Mohammadi M (2022). Application of undecanedicarboxylic acid to prepare cross-linked enzymes (CLEs) of *Rhizomucor miehei* lipase (RML); Selective enrichment of polyunsaturated fatty acids. J Mol Catal.

[CR3] Akil E, da Adejanildo S, Pereira TE-B, Amaral PFF, Torres AG (2020). Efficient production of bioactive structured lipids by fast acidolysis catalyzed by *Yarrowia lipolytica* lipase, free and immobilized in chitosan-alginate beads, in solvent-free medium. Int J Biol Macromol.

[CR4] Aldai N, de Renobales M, Barron LJR, Kramer JKG (2013). What are the trans fatty acids issues in foods after discontinuation of industrially produced trans fats? Ruminant products, vegetable oils, and synthetic supplements. Eur J Lipid Sci Technol.

[CR5] Almeida FLC, Castro MPJ, Travália BM, Forte MBS (2021). Trends in lipase immobilization: bibliometric review and patent analysis. Process Biochem.

[CR6] Arnold FH (2018). Directed evolution: bringing new chemistry to life. Angew Chem Int Ed Engl.

[CR7] Baek M, DiMaio F, Anishchenko I, Dauparas J, Ovchinnikov S, Lee GR, Wang J, Cong Q, Kinch LN, Schaeffer RD, Millán C, Park H, Adams C, Glassman CR, DeGiovanni A, Pereira JH, Rodrigues AV, van Dijk AA, Ebrecht AC, Opperman DJ, Sagmeister T, Buhlheller C, Pavkov-Keller T, Rathinaswamy MK, Dalwadi U, Yip CK, Burke JE, Garcia KC, Grishin NV, Adams PD, Read RJ, Baker D (2021). Accurate prediction of protein structures and interactions using a three-track neural network. Science.

[CR8] Bell EL, Smithson R, Kilbride S, Foster J, Hardy FJ, Ramachandran S, Tedstone AA, Haigh SJ, Garforth AA, Day PJR, Levy C, Shaver MP, Green AP (2022). Directed evolution of an efficient and thermostable PET depolymerase. Nat Catal.

[CR9] Bergamo P, Luongo D, Miyamoto J, Cocca E, Kishino S, Ogawa J, Tanabe S, Rossi M (2014). Immunomodulatory activity of a gut microbial metabolite of dietary linoleic acid, 10-hydroxy-cis-12-octadecenoic acid, associated with improved antioxidant/detoxifying defences. J Funct Foods.

[CR10] Bernal C, Rodríguez K, Martínez R (2018). Integrating enzyme immobilization and protein engineering: an alternative path for the development of novel and improved industrial biocatalysts. Biotechnol Adv.

[CR11] Biermann U, Bornscheuer UT, Feussner I, Meier MAR, Metzger JO (2021). Fatty acids and their derivatives as renewable platform molecules for the chemical industry. Angew Chem Int Ed Engl.

[CR12] Bornscheuer UT (2014). Enzymes in lipid modification: past achievements and current trends. Eur J Lipid Sci Technol.

[CR13] Bornscheuer UT (2018). Chapter 1—enzymes in lipid modification: an overview. Lipid modification by enzymes and engineered microbes.

[CR14] Bornscheuer UT, Hauer B, Jaeger KE, Schwaneberg U (2019). Directed evolution empowered redesign of natural proteins for the sustainable production of chemicals and pharmaceuticals. Angew Chem Int Ed Engl.

[CR15] Cao J, Wu R, Zhu F, Dong QH, Su E (2022). How to improve the efficiency of biocatalysis in non-aqueous pure deep eutectic solvents: a case study on the lipase-catalyzed transesterification reaction. Biochem Eng J.

[CR16] Chen G, Khan IM, He WS, Li YX, Jin P, Campanella OH, Zhang HH, Huo YR, Chen Y, Yang HQ, Miao M (2022). Rebuilding the lid region from conformational and dynamic features to engineering applications of lipase in foods: Current status and future prospects. Compr Rev Food Sci Food Saf.

[CR17] Chow JY, Nguyen GKT (2022). Rational design of lipase ROL to increase its thermostability for production of structured TAGs. Int J Mol Sci.

[CR18] Damnjanović J, Matsunaga N, Adachi M, Nakano H, Iwasaki Y (2018). Facile enzymatic synthesis of phosphatidylthreonine using an engineered Phospholipase D. Eur J Lipid Sci Technol.

[CR19] de Rodrigues CA, Barbosa MS, dos Santos JCB, Lisboa MC, Souza RL, Pereira MM, Lima ÁS, Soares CMF (2021). Computational and experimental analysis on the preferential selectivity of lipases for triglycerides in Licuri oil. Bioprocess Biosyst Eng.

[CR20] Enespa CP, Singh DP (2022). Sources, purification, immobilization and industrial applications of microbial lipases: an overview. Crit Rev Food Sci Nutr.

[CR21] Eser BE, Poborsky M, Dai R, Kishino S, Ljubic A, Takeuchi M, Jacobsen C, Ogawa J, Kristensen P, Guo Z (2020). Rational engineering of hydratase from lactobacillus acidophilus reveals critical residues directing substrate specificity and regioselectivity. ChemBioChem.

[CR22] Ge FY, Chen G, Qian MJ, Xu C, Liu J, Cao JQ, Li XC, Hu D, Xu YS, Xin Y, Wang DL, Zhou J, Shi H, Tan ZB (2023). Artificial intelligence aided lipase production and engineering for enzymatic performance improvement. J Agric Food Chem.

[CR23] Hayashi D, Mouchlis VD, Dennis EA (2021). Omega-3 versus Omega-6 fatty acid availability is controlled by hydrophobic site geometries of phospholipase A2s. J Lipid Res.

[CR24] Hu R, Cui R, Tang Q, Lan D, Wang F, Wang Y (2021). Enhancement of phospholipid binding and catalytic efficiency of *Streptomyces klenkii* Phospholipase D by increasing hydrophobicity of the active site loop. J Agric Food Chem.

[CR25] Huang J, Dai S, Chen X, Xu L, Yan J, Yang M, Yan Y (2023). Alteration of chain-length selectivity and thermostability of *Rhizopus oryzae* lipase via virtual saturation mutagenesis coupled with disulfide bond design. Appl Environ Microbiol.

[CR26] Inoue A, Adachi M, Damnjanović J, Nakano H, Iwasaki Y (2016). Direct enzymatic synthesis of 1-phosphatidyl-β-D-glucose by engineered phospholipase D. ChemistrySelect.

[CR27] Kuchner OAF (1997). Directed evolutionof enzyme catalysts. Trends Biotechnol.

[CR28] Lan D, Zhao G, Holzmann N, Yuan S, Wang J, Wang Y (2021). Structure-guided rational design of a mono- and diacylglycerol lipase from Aspergillus oryzae: a single residue mutant increases the hydrolysis ability. J Agric Food Chem.

[CR29] Lee WJ, Zhang Z, Lai OM, Tan CP, Wang Y (2020). Diacylglycerol in food industry: synthesis methods, functionalities, health benefits, potential risks and drawbacks. Trends Food Sci Technol.

[CR30] Li D, Han T, Xue J, Xu W, Xu J, Wu Q (2021). Engineering fatty acid photodecarboxylase to enable highly selective decarboxylation of trans fatty acids. Angew Chem Int Ed Engl.

[CR31] Li L, Mao X, Deng F, Wang Y, Wang F (2022). Improving both the thermostability and catalytic efficiency of phospholipase d from moritella sp. JT01 through disulfide bond engineering strategy. Int J Mol Sci.

[CR32] Li L, Wu W, Deng Z, Zhang S, Guan W (2022). Improved thermostability of lipase Lip2 from Yarrowia lipolytica through disulfide bond design for preparation of medium-long-medium structured lipids. LWT.

[CR33] Li Z, Meng S, Nie K, Schwaneberg U, Davari MD, Xu H, Ji Y, Liu L (2022). Flexibility regulation of loops surrounding the tunnel entrance in cytochrome P450 enhanced substrate access substantially. ACS Catal.

[CR34] Liu Y, Liu Q, Krivoruchko A, Khoomrung S, Nielsen J (2020). Engineering yeast phospholipid metabolism for de novo oleoylethanolamide production. Nat Chem Biol.

[CR35] Lovelock SL, Crawshaw R, Basler S, Levy C, Baker D, Hilvert D, Green AP (2022). The road to fully programmable protein catalysis. Nature.

[CR36] Lu H, Diaz DJ, Czarnecki NJ, Zhu C, Kim W, Shroff R, Acosta DJ, Alexander BR, Cole HO, Zhang Y, Lynd NA, Ellington AD, Alper HS (2022). Machine learning-aided engineering of hydrolases for PET depolymerization. Nature.

[CR37] Madhavan A, Arun KB, Binod P, Sirohi R, Tarafdar A, Reshmy R, Kumar Awasthi M, Sindhu R (2021). Design of novel enzyme biocatalysts for industrial bioprocess: Harnessing the power of protein engineering, high throughput screening and synthetic biology. Bioresour Technol.

[CR38] Maldonado MR, Alnoch RC, Marques J, de Almeida L, dos Santos A, Andretta AT, del Pilar R, Ropaín C, Maltempi E, de Souza D, Mitchell A, Krieger N (2021). Key mutation sites for improvement of the enantioselectivity of lipases through protein engineering. Biochem Eng J.

[CR39] Markel U, Lanvers P, Sauer DF, Wittwer M, Dhoke GV, Davari MD, Schiffels J, Schwaneberg U (2021). A Photoclick-based high-throughput screening for the directed evolution of decarboxylase OleT. Chem Eur J.

[CR40] Martins PA, Trobo-Maseda L, Lima FA, de Morais JWG, De Marco JL, Salum TFC, Guisán JM (2022). Omega-3 production by fish oil hydrolysis using a lipase from *Burkholderia gladioli* BRM58833 immobilized and stabilized by post-immobilization techniques. Biochem Biophys Rep.

[CR41] Mazurenko S, Prokop Z, Damborsky J (2019). Machine learning in enzyme engineering. ACS Catal.

[CR42] McDaniel MA, Maier SF, Einstein GO (2003). "Brain-specific" nutrients: a memory cure?. Nutrition.

[CR43] Micha R, Mozaffarian D (2009). Trans fatty acids: effects on metabolic syndrome, heart disease and diabetes. Nat Rev Endocrinol.

[CR44] Nielsen J, Keasling JD (2016). Engineering cellular metabolism. Cell.

[CR45] Ogawa J (2015). New lipid science in our inner ecosystem. Eur J Lipid Sci Technol.

[CR46] Pacheco BJS, Domingues O, Reina MP, de Baptista Álvaro, Neto GS, Andrade S, Veloso A, de Paula,  (2022). Improved synthesis of dietary triglycerides by using lipase supported on clay carriers. Biotechnol J.

[CR47] Prabhavathi Devi BLA, Gangadhar KN, Prasad RBN, Sugasini D, Rao YPC, Lokesh BR (2018). Nutritionally enriched 1,3-diacylglycerol-rich oil: low calorie fat with hypolipidemic effects in rats. Food Chem.

[CR48] Qi N, Liu J, Song W, Liu J, Gao C, Chen X, Guo L, Liu L, Wu J (2022). Rational design of phospholipase d to improve the transphosphatidylation activity for phosphatidylserine synthesis. J Agric Food Chem.

[CR49] Qu G, Li A, Acevedo-Rocha CG, Sun Z, Reetz MT (2020). The crucial role of methodology development in directed evolution of selective enzymes. Angew Chem Int Ed Engl.

[CR50] Rathi PC, Fulton A, Jaeger K-E, Gohlke H (2016). Application of rigidity theory to the thermostabilization of lipase a from bacillus subtilis. Plos Comput Biol.

[CR51] Reetz MT, Carballeira JD (2007). Iterative saturation mutagenesis (ISM) for rapid directed evolution of functional enzymes. Nat Protoc.

[CR52] Reetz MT, Bocola M, Carballeira JD, Zha D, Vogel A (2005). Expanding the range of substrate acceptance of enzymes: combinatorial active-site saturation test. Angew Chem Int Ed Engl.

[CR53] Samantha A, Damnjanović J, Iwasaki Y, Nakano H, Vrielink A (2021). Structures of an engineered phospholipase D with specificity for secondary alcohol transphosphatidylation: insights into plasticity of substrate binding and activation. Biochem J.

[CR54] Soni S (2022). Trends in lipase engineering for enhanced biocatalysis. Biotechnol Appl Biochem.

[CR55] Soni S, Sathe SS, Sheth RR, Tiwari P, Vadgama R-KN, Odaneth AA, Lali AM, Chandrayan SK (2019). N-terminal domain replacement changes an archaeal monoacylglycerol lipase into a triacylglycerol lipase. Biotechnol Biofuels.

[CR56] Sulciner ML, Serhan CN, Gilligan MM, Mudge DK, Chang J, Gartung A, Lehner KA, Bielenberg DR, Schmidt B, Dalli J, Greene ER, Gus-Brautbar Y, Piwowarski J, Mammoto T, Zurakowski D, Perretti M, Sukhatme VP, Kaipainen A, Kieran MW, Huang S, Panigrahy D (2018). Resolvins suppress tumor growth and enhance cancer therapy. J Exp Med.

[CR57] Sun Z, Liu Q, Qu G, Feng Y, Reetz MT (2019). Utility of B-factors in protein science: interpreting rigidity, flexibility, and internal motion and engineering thermostability. Chem Rev.

[CR58] Tang Q, Lan D, Yang B, Khan FI, Wang Y (2017). Site-directed mutagenesis studies of hydrophobic residues in the lid region of T1 lipase. Eur J Lipid Sci Technol.

[CR59] Varadi M, Anyango S, Deshpande M, Nair S, Natassia C, Yordanova G, Yuan D, Stroe O, Wood G, Laydon A, Zidek A, Green T, Tunyasuvunakool K, Petersen S, Jumper J, Clancy E, Green R, Vora A, Lutfi M, Figurnov M, Cowie A, Hobbs N, Kohli P, Kleywegt G, Birney E, Hassabis D, Velankar S (2022). AlphaFold protein structure database: massively expanding the structural coverage of protein-sequence space with high-accuracy models. Nucleic Acids Res.

[CR60] Verdasco-Martín CM, Garcia-Verdugo E, Porcar R, Fernandez-Lafuente R, Otero C (2018). Selective synthesis of partial glycerides of conjugated linoleic acids via modulation of the catalytic properties of lipases by immobilization on different supports. Food Chem.

[CR61] Verger R (1997). ‘Interfacial activation’ of lipases: facts and artifacts. Trends Biotechnol.

[CR62] Wang S, Xu Y, Yu X-W (2021). Propeptide in Rhizopus chinensis lipase: new insights into its mechanism of activity and substrate selectivity by computational design. J Agric Food Chem.

[CR63] Wang Z, Wen J, Zhang J, Deng J, Zhuang W, Liu J, Wang Z, Rao Y, Zhu Y, Ying H (2023). Atomic insights into the mechanism of trace water influence on lipase catalysis in organic media. Chem Eng J.

[CR64] Watson JL, Juergens D, Bennett NR, Trippe BL, Yim J, Eisenach HE, Ahern W, Borst AJ, Ragotte RJ, Milles LF, Wicky BIM, Hanikel N, Pellock SJ, Courbet A, Sheffler W, Wang J, Venkatesh P, Sappington I, Torres SV, Lauko A, De Bortoli V, Mathieu E, Ovchinnikov S, Barzilay R, Jaakkola TS, DiMaio F, Baek M, Baker D (2023). De novo design of protein structure and function with RFdiffusion. Nature.

[CR65] Woo J-M, Kang Y-S, Lee S-M, Park S, Park J-B (2022). Substrate-binding site engineering of Candida antarctica Lipase B to improve selectivity for synthesis of 1-monoacyl-sn-glycerols. Biotechnol Bioprocess Eng.

[CR66] Wu C, Hong B, Jiang S, Luo X, Lin H, Zhou Y, Wu J, Yue X, Shi H, Wu R (2022). Recent advances on essential fatty acid biosynthesis and production: clarifying the roles of Δ12/Δ15 fatty acid desaturase. Biochem Eng J.

[CR67] Wu L, Qin L, Nie Y, Xu Y, Zhao YL (2022). Computer-aided understanding and engineering of enzymatic selectivity. Biotechnol Adv.

[CR68] Wu S, Xiang C, Zhou Y, Khan MSH, Liu W, Feiler CG, Wei R, Weber G, Hohne M, Bornscheuer UT (2022). A growth selection system for the directed evolution of amine-forming or converting enzymes. Nat Commun.

[CR69] Xu Q, Tang Q, Xu Y, Wu J, Mao X, Li F, Wang S, Wang Y (2023). Biotechnology in future food lipids: opportunities and challenges. Annu Rev Food Sci Technol.

[CR70] Yang Y, Arnold FH (2021). Navigating the unnatural reaction space: directed evolution of heme proteins for selective Carbene and Nitrene transfer. Acc Chem Res.

[CR71] Yang Y, Wang J, Yang B, Lan D, Wang Y (2022). Possible charged residue switch for acylglycerol selectivity of lipase MAS1. Appl Biochem Biotechnol.

[CR72] Yi J-J, Heo S-Y, Ju J-H, Oh B-R, Son WS, Seo J-W (2020). Synthesis of 13R,20-dihydroxy-docosahexaenoic acid by site-directed mutagenesis of lipoxygenase derived from Oscillatoria nigro-viridis PCC 7112. Biochem Biophys Res Commun.

[CR73] Yore Mark M, Syed I, Moraes-Vieira Pedro M, Zhang T, Herman Mark A, Homan Edwin A, Patel Rajesh T, Lee J, Chen S, Peroni Odile D, Dhaneshwar Abha S, Hammarstedt A, Smith U, McGraw Timothy E, Saghatelian A, Kahn Barbara B (2014). Discovery of a class of endogenous mammalian lipids with anti-diabetic and anti-inflammatory effects. Cell.

[CR74] Yu D, Wang JB, Reetz MT (2019). Exploiting designed oxidase-peroxygenase mutual benefit system for asymmetric cascade reactions. J Am Chem Soc.

[CR75] Zeng W, Guo L, Xu S, Chen J, Zhou J (2020). High-throughput screening technology in industrial biotechnology. Trends Biotechnol.

[CR76] Zhang H, Chu W, Sun J, Liu Z, Huang WC, Xue C, Mao X (2019). Combining cell surface display and DNA-shuffling technology for directed evolution of streptomyces phospholipase d and synthesis of phosphatidylserine. J Agric Food Chem.

[CR77] Zhang TT, Xu J, Wang YM, Xue CH (2019). Health benefits of dietary marine DHA/EPA-enriched glycerophospholipids. Prog Lipid Res.

[CR78] Zhang M, Li Q, Lan X, Li X, Zhang Y, Wang Z, Zheng J (2020). Directed evolution of Aspergillus oryzae lipase for the efficient resolution of (R, S)-ethyl-2-(4-hydroxyphenoxy) propanoate. Bioprocess Biosyst Eng.

[CR79] Zhang Z, Chen M, Xu W, Zhang W, Zhang T, Guang C, Mu W (2020). Microbial phospholipase D: Identification, modification and application. Trends Food Sci Technol.

[CR80] Zhang H, Secundo F, Sun J, Mao X (2022). Advances in enzyme biocatalysis for the preparation of functional lipids. Biotechnol Adv.

[CR81] Zhang Y, Dai P, Liu R, Liu W, Xiao A, Li J, Li G, Liu J (2022). Rational engineering of phospholipase C from Bacillus cereus HSL3 for simultaneous thermostability and activity improvement. J Biotechnol.

[CR82] Zheng WL, Pu ZJ, Xiao LX, Xu G, Yang LR, Yu HR, Wu JP (2023). Mutability-landscape-guided engineering of l-threonine aldolase revealing the prelog rule in mediating diastereoselectivity of C-C bond formation. Angew Chem Int Ed Engl.

[CR83] Zhu L, Fang SZ, Liu WW, Zhang H, Zhang YQ, Xie ZH, Yang PY, Wan JC, Gao BY, Yu L (2023). The triacylglycerol structure and composition of a human milk fat substitute affect the absorption of fatty acids and calcium, lipid metabolism and bile acid metabolism in newly-weaned Sprague-Dawley rats. Food Funct.

[CR84] Zong L, Zhang Y, Shao Z, Ljubic A, Jacobsen C, Gao R, Eser BE, Wang Y, Guo Z (2023). Selective and sustainable production of sub-terminal hydroxy fatty acids by a self-sufficient CYP102 enzyme from bacillus amyloliquefaciens. ChemBioChem.

[CR85] Zorn K, Oroz-Guinea I, Brundiek H, Bornscheuer UT (2016). Engineering and application of enzymes for lipid modification, an update. Prog Lipid Res.

[CR86] Zorn K, Oroz-Guinea I, Brundiek H, Dörr M, Bornscheuer UT (2018). Alteration of chain length selectivity of candida antarctica lipase A by semi-rational design for the enrichment of Erucic and Gondoic fatty acids. Adv Synth Catal.

[CR87] Zorn K, Oroz-Guinea I, Bornscheuer UT (2019). Strategies for enriching erucic acid from Crambe abyssinica oil by improved Candida antarctica lipase A variants. Process Biochem.

[CR88] Zou XQ, Ye LF, He XC, Wu SB, Zhang H, Jin QZ (2020). Preparation of DHA-rich medium- and long-chain triacylglycerols by lipase-catalyzed acidolysis of microbial oil from *Schizochytrium sp* with medium-chain fatty acids. Appl Biochem Biotechnol.

